# Labour migration of Polish nurses: a questionnaire survey conducted with the Computer Assisted Web Interview technique

**DOI:** 10.1186/s12960-016-0124-9

**Published:** 2016-06-30

**Authors:** Rafał Szpakowski, Patrycja W. Zając, Grażyna Dykowska, Zofia Sienkiewicz, Anna Augustynowicz, Aleksandra Czerw

**Affiliations:** Department of Public Health, Faculty of Health Sciences, Medical University of Warsaw, Banacha 1a (unit F), 02-097 Warsaw, Poland; Department of Clinical Nursing, Faculty of Health Sciences, Medical University of Warsaw, Warsaw, Poland; Department of Public Nursing, Faculty of Health Sciences, Medical University of Warsaw, Warsaw, Poland

**Keywords:** Healthcare workers, Health policy, Labour migration, Nurse migration, Polish nurses, Questionnaire survey, Work satisfaction

## Abstract

**Background:**

According to data from the Organisation for Economic Cooperation and Development, Poland has one of the lowest numbers of nurses (5.2) per 1000 inhabitants among 28 EU countries. The migration of nurses from Poland has particular importance in the context of scarce human resources in this professional group, especially given the increasingly ageing population in European societies, which will entail an increased demand for nursing and care services. The aim of the study was to obtain information on the intentions of Polish nurses to migrate for work to other countries in the European region.

**Methods:**

The study included 581 nurses, professionally active in Poland over the duration of the study. The Computer Assisted Web Interview technique was used to collect data. Nurses filled in a web-based questionnaire that was available from December 5, 2011, to March 5, 2012. The choice of respondents for the sample was based on the availability of data. An invitation to participate in the study could be viewed on selected websites from the Portal of Nurses and Midwives, the Supreme Chamber of Nurses and Midwives, and the Polish Nursing Society. The survey questionnaire was designed by the authors, which served as the primary research tool.

**Results:**

Nearly one in three respondents intended to leave Poland for professional reasons. Overall, 12.4 % of respondents had already worked as a nurse abroad. The main destinations for migration included Germany, followed by England and Norway. The intended length of stay abroad ranged from 2–5 years.

**Conclusions:**

In the studied group of Polish nurses, there was great interest in seeking employment abroad. Nurses tend to go abroad mostly for long-term, repeated periods to the wealthiest countries nearest to Poland. In view of the low level of human resources in the Polish nursing sector, the migration of Polish nurses will probably have crucial implications for the quality of healthcare services in Poland in the coming years. Given the methodology applied, study findings refer solely to the study group.

## Background

Common scientific literature databases, such as PubMed, as well as statistical data from the Central Statistical Office in Poland do not contain information on the subject of Polish nurse migration. Estimates with respect to the scale of the migration of Polish nurses abroad are based solely on the Register of the Supreme Chamber of Nurses and Midwives, which contains a record of the number of certificates issued as recognition of professional qualifications as required by other European Union (EU) countries [1]. Based on this method of nurse migration evaluation, it can be assumed that, by the end of 2013, 7 % of nurses employed as of March 21, 2013, left Poland for professional reasons [2]. Problems related to the evaluation of the actual scale of Polish nurse migration arise from two phenomena. Firstly, not all nurses who apply for their certificate actually collect it, and secondly, from those that do, not all decide to finally migrate and some undertake work initially below their qualifications, for example, as nursing assistants or caretakers of elderly people, due to an insufficient command of the local language.

The migration of nurses has had an adverse impact on the already human resource scarce Polish nursing sector. According to Organisation for Economic Cooperation and Development data [3], Poland has one of the lowest numbers of nurses (5.2) per 1000 inhabitants among the 28 EU countries [3, 4]. The migration of nurses makes the management of human resources in healthcare difficult due to the absence of a reliable method to estimate this phenomenon. Polish authorities have hardly any knowledge of the number of nurses leaving the country. There are no instruments that monitor or regulate the flow of human capital from Poland to other European and non-European countries in the area of healthcare.

The migration of nurses from Poland is particularly advantageous for the developed countries of Western Europe, which have an ever increasing need for nurses that satisfy European qualification standards given their increasing ageing population [5]. Poland can be a potential and significant source of nursing personnel for these countries since, being a member state of the EU, it prepares nursing personnel whose qualifications are automatically recognized in all member states [6]. Further, the Polish healthcare sector has experienced long-lasting problems related to its management [7], and therefore there is a real threat from the loss of Polish nursing resources to migration in the coming years.

The aim of the present study was to obtain information on the intentions of Polish nurses to migrate for work to other countries in the European region. In particular, the study aims to identify (1) directions of intended migration, (2) the duration of intended stay abroad, (3) motives for leaving the country, (4) barriers to the decision to migrate, (5) financial aspirations of the respondents related to work abroad, and to determine the nature of work undertaken by the respondents abroad in terms of (6) the place of work and (7) the work position (lower, consistent with, or higher qualifications).

## Methods

The study included 581 professionally active nurses working in Poland at the time the survey was conducted. Characteristics of the study group are detailed in Table 1.

**Table 1:** Characteristics of the study group (*n* = 581)

Variables	“Do you intend to emigrate from Poland to another country to take up work there?” *n* (%)		
	Yes	No	Total
Total	172 (100)	409 (100)	581 (100)
Please, specify whether you have worked abroad as a nurse:			
Yes	44 (26)	28 (7)	72 (12)
No	110 (64)	361 (88)	471 (81)
Occupation other than a nurse [caretakers of the elderly (*n* = 26), nursing assistant (*n* = 6), seasonal work or catering facilities (*n* = 6)]	18 (10)	20 (5)	38 (7)
Please, indicate one major phenomenon which makes practising the profession of a nurse in Poland hardly attractive			
Low remuneration in comparison with great responsibility and social expectations	132 (77)	291 (71)	423 (73)
Great mental and physical load caused by an insufficient number of personnel in relation to the number of patients	24 (14)	63 (16)	87 (15)
Low level of social respect for people practising the profession of a nurse	16 (9)	55 (15)	71 (12)
Sex			
Women	149 (87)	386 (94)	535 (92)
Men	23 (13)	23 (6)	46 (8)
Age, years			
20–25	21 (12)	19 (5)	40 (7)
26–31	31 (18)	38 (9)	69 (12)
32–37	31 (18)	66 (16)	97 (17)
38–43	43 (25)	131 (32)	174 (30)
44–49	27 (16)	96 (24)	123 (21)
50+	19 (11)	59 (14)	78 (13)
Place of residence			
Village	22 (13)	86 (21)	108 (19)
City with less than 20 k inhabitants	22 (13)	56 (14)	78 (13)
City with less than 50 k inhabitants	18 (10)	63 (15)	81 (14)
City with 50–100 k inhabitants	25 (14)	53 (13)	78 (13)
City with 500 k to 1 m inhabitants	41 (24)	82 (20)	123 (21)
City above 1 m inhabitants	44 (26)	69 (17)	113 (20)
Marital status			
Married	97 (56)	302 (74)	399 (68)
Informal relationship	43 (25)	54 (13)	97 (17)
No relationship	32 (19)	53 (13)	85 (15)
Net earnings (PLN) per month ($US 1 = 3.7 PLN)			
Under 2,000	55 (32)	81 (20)	136 (23)
2,000–3,000	88 (51)	219 (54)	307 (53)
3,000–4,000	20 (12)	71 (17)	91 (16)
4,000–5,000	7 (4)	29 (7)	36 (6)
5,000 or above	2 (1)	8 (2)	10 (2)
Assessment of own material standing			
Very bad	9 (5)	6 (2)	15 (2)
Bad	55 (32)	41 (10)	96 (17)
Satisfactory	83 (48)	185 (45)	268 (46)
Good	24 (14)	161 (39)	185 (32)
Very good	1 (1)	16 (4)	17 (3)
Number of workplaces			
One	108 (63)	281 (69)	389 (67)
Two	50 (29)	97 (24)	147 (25)
Three	13 (7)	28 (6)	41 (7)
Four or more	1 (1)	3 (1)	4 (1)
Place of employment (multiple choice question)			
Public hospital	135 (79)	271 (66)	406 (70)
Not public hospital	21 (12)	49 (12)	70 (12)
Public primary healthcare	16 (9)	41 (10)	57 (10)
Not public primary healthcare	22 (13)	64 (16)	86 (15)
Working years (mean = 17.66; standard deviation = 9.88; median = 19; mode = 20; *n* mode = 37)			
Up to one year	13 (8)	15 (4)	28 (5)
1–5	39 (23)	38 (9)	77 (13)
6–10	15 (9)	33 (8)	48 (8)
11–15	23 (13)	50 (12)	73 (13)
16–20	29 (17)	77 (19)	106 (18)
21–25	23 (13)	85 (21)	108 (19)
26–30	18 (10)	65 (16)	83 (14)
31–35	9 (5)	39 (10)	48 (8)
36–40	1 (1)	7 (2)	8 (1)
Hospital ward profile (multiple choice question)	143 (100)	303 (100)	446 (100)
ICU	33 (23)	47 (16)	80 (18)
Surgical, including cardiac surgery, oncological surgery, neurosurgery, trauma and general surgery	19 (13)	37 (12)	56 (13)
Internal medicine	15 (10)	31 (10)	46 (10)
Cardiology	11 (8)	22 (7)	33 (7)
Operating theatre	9 (6)	22 (7)	31 (7)
Paediatric	9 (6)	24 (8)	33 (7)
Other	120 (84)	47 (16)	167 (37)

The Computer Assisted Web Interview technique was used to collect data. Nurses filled in a web-based questionnaire, which was available from December 5, 2011, to March 5, 2012. The collection of questionnaires was completed after three months because the number of survey responses had dropped to below one per week. The choice of respondents for the sample was based on their availability, i.e. the respondents themselves decided whether they wanted to participate in the study. An invitation to the study, along with the survey page address, was published on the front page of the websites of the Portal of Nurses and Midwives, the Supreme Chamber of Nurses and Midwives, the Polish Nursing Society, and the Health Market Portal, which were accessible to nurses and nursing-related personnel, as well as on the web-pages of the District Nursing Chambers in Warsaw, Krosno, Rzeszów, Poznań, and Białystok.

The research tool used was a purpose-designed three-part questionnaire. The first part, to be filled by all respondents, contained questions concerning their socio-demographic status. The second part contained questions related to their migration decision — respondents who did not intend to or did not migrate gave reasons for their decision and finalized the questionnaire at this point. Respondents who declared an intention to migrate moved on to the third part of the questionnaire and answered questions aimed at determining the motives underlying their desire to migrate, as well as the country of migration and the duration. The collected data was analysed using descriptive statistics, including proportions, mean, median, and mode, where possible.

The respondents were informed that the study was carried out by the Medical University of Warsaw and were familiarized with the study purpose. Each study subject was informed that the results obtained would be used for research purposes only. All individuals included in the study were adults. Ethics approval was not necessary for this study and therefore the authors have not approached an ethics committee for approval.

### Inclusion and exclusion criteria

Overall, 119/700 (17.0 %) questionnaires were excluded from the study since they were completed by unemployed nurses or nurses employed abroad at the time of the study (the aim of the study was to analyse the phenomenon of migration with respect to current human resources in the Polish nursing sector), were incomplete (to less than 80 %), or logically inconsistent due to contradictory answers determined on the basis of cross-questions (e.g. a respondent gives a reply about the place of work (hospital) and in another reply denies working in a hospital).

## Results

Overall, 29.6 % (172/581) of respondents intended to emigrate from Poland for professional reasons and 12.4 % (72/581) had already worked abroad as a nurse. Further, 72.8 % (423/581) believed that it is disadvantageous to practise the profession of a nurse in Poland due to the inadequacy of earnings against the scope of their duties (Table 1).

The lack of intention to take up work abroad was explained by most respondents in terms of family-related considerations (Table 2). Economic considerations constituted the most common reason for migration among respondents declaring the intention to go abroad for professional reasons. The main destinations indicated were Germany, followed by England. The intended duration of stay abroad most frequently ranged from 2–5 years. The majority of respondents expected to undertake work abroad consistent with their qualifications in hospital or nursing homes and to send money to Poland. The expected earnings per month (in PLN) abroad ranged from 6,500–10,000 ($US 1,722–2,650; Table 3). Among short-term (non-permanent) migrants, the majority intended to re-migrate to the same country in which they had previously worked (Table 4).

**Table 2:** Questionnaire findings – replies by non-migrating respondents (*n* = 409)

Number of questions/content of the questions and answers, n (%)	
4. Please, indicate one major reason why you do not intend to migrate from Poland	
Family situation does not allow it	154 (38)
I find my present material and professional situation satisfactory	69 (17)
I don’t know foreign languages	68 (17)
I am afraid I will not be able to cope in a new cultural and language environment.	52 (13)
Age	22 (5)
Other	
[My state of health does not allow it (9); Participation in the reform of Polish nursing (3); I love my country and I want to support it (7); Fear of losing the professional position (3); I don’t have a university diploma (12); My husband earns a lot (1); I already worked for 7 years (1); Mental loss cannot be offset by material gains (1); Complicated formalities making legal work abroad difficult to undertake (5); Hope for better earnings (2)	44 (10)
Data gaps	–

**Table 3:** Questionnaire findings – replies given by migrating respondents (n = 172)

Number of questions/content of the questions and answers, n (%)		
1. Please, indicate one major element which made you consider the country of destination attractive		
	High salaries	84 (49)
	Better living conditions (better support from the state)	38 (22)
	Better working conditions and professional development	33 (19)
	Higher public respect for the profession of a nurse	14 (8)
	Valuable experience	3 (2)
	Data gaps	–
2. Please, indicate the country you want to migrate to		
	Germany	55 (32)
	England	32 (19)
	Norway	20 (12)
	Switzerland	18 (11)
	Italy	11 (6)
	USA	9 (5)
	Other [Afghanistan (1), Austria (2), Belgium (3), Czech Republic (1), Denmark (1), Finland (2), the Netherlands (1), Spain (2), Ireland (5), Canada (2), Libya (1), Sweden (5), Australia (1)]	27 (15)
	Data gaps	–
3. Please, indicate the duration of your intended stay abroad		
	One year or less	33 (19)
	From 2–5 years	70 (41)
	Above 5 years	3 (2)
	Permanently	64 (37)
	Data gaps	2 (1)
4. Please, complete the sentence: “I intend to undertake abroad work …”		
	…requiring lower qualifications than I have because I will work as … [nursing assistance (3), elderly carer (14)]	17 (9.5)
	… consistent with my qualifications and skills because I will work as … [nurse (152), manager (2)]	154 (90)
	…requiring higher qualifications than I have because I will work as … [teacher (1)]	1 (0.5)
	Data gaps	–
5. Please, complete the sentence: “My first place of work abroad will be …”		
	Hospital	86 (50)
	Nursing home	47 (27)
	Home care of an elderly person	27 (16)
	Other [social welfare home (4), care of wounded soldiers (1), training company (1), hospice (palliative care) (3), I cannot specify (3)]	12 (7)
	Data gaps	–
6. Please, specify whether you intend to send part of your earnings abroad to Poland		
	Yes	103 (60)
	No	69 (40)
	Data gaps	–
7. Please, specify what net remuneration per month you expect to get from one place of work whether under an employment contract or under a civil law contract abroad (in PLN) [$US 1 = 3.7 PLN]		
	between 3,000 and 4,500	9 (5)
	between 5,000 and 6,000	31 (18)
	between 6,500 and 8,000	47 (27)
	between 8,500 and 10,000	37 (22)
	between 11,000 and 15,000	31 (18)
	between 16,000 and 20,000	9 (5)
	I don’t know	8 (5)
	Data gaps	–

**Table 4:** Questionnaire findings – replies of respondents migrating for a specified period of time (*n* = 106)

Number of questions/content of the questions and answers, *n* (%)	
What are your plans for after the period of migration?	
I intend to return to my home country for good, my stay abroad was but an episode	30 (28)
I intend to return to my home country and migrate again, this time to another country	9 (8.5)
I am not returning to my country but migrating to another one	9 (8.5)
I intend to return to the home country for some time only to migrate to the same country as before	58 (55)
Data gaps	–

## Discussion

There is no doubt that Poland’s integration with the EU opened new opportunities for medical personnel whose qualifications and skills have come to be particularly appreciated and desired [8]. These opportunities are a sharp contrast against the unfavourable demographic processes and trends in the Polish population [9]. Moreover, the ageing of professionals in the European medical market is becoming an issue [10] and emerging shortages in human resources are a main factor determining the future of health policy both in Poland and throughout the EU [11]. Hence, there is a well justified need to study the patterns of remuneration-oriented labour mobility among professionally active nurses who intend to seek employment abroad.

The determination and knowledge of patterns related to remuneration-oriented mobility of Polish nurses abroad is of essential importance, especially when considering the requirement to satisfy the future needs of an ageing society. It should be noted that the subject of migration among Polish nurses does not seem to be in the scope of interest of political decision makers. This is substantiated by a lack of reliable data on the subject of migration among healthcare workers in general and not only nurses, which, in turn, makes the development of evidence-based policy impossible. Moreover, Polish scientific circles seem to have totally neglected the issue as no original study on the migration of professionally active Polish nurses can be found among scientific publications. Therefore, no previous study findings in this area are available for referral.

The present study estimates a scale of migration at the level of approximately 30 % of the study group. This seems to be a significant percentage given the fact that data proceeding from the Supreme Chamber of Nurses and Midwives shows that interest in migration in the years 2004–2013 amounted to around 7 % of nurses employed in a basic place of work [1, 2]. It should be noted, however, that the data referred to is in fact based only on a declaration, while the fact of migration is not registered. Further, differences may have arisen by the fact that the question did not specify the moment of migration (e.g. year of leaving the country) and therefore the result should be treated as an overall interest in migration, not limited by the perspective of time. Thus, one third of the current respondents are expected to migrate at least once in their lifetime.

Although migration was intended by a minority of respondents, attention should be given to the trend, which is likely to gain momentum. This should provide a warning signal for health policymakers, given the high percentage of the respondents’ answers confirming earlier employment outside Poland. As in the case of future migration, the scale of past migration seems significant, indicating a high mobility abroad among the respondents [1].

According to Lee [12], it is the push factors present in the migrant’s home country and the pull factors in the country of migration that influence the decision to migrate, along with personal factors, which the theory also mentions. Generalizing, one might say that in the case of professional migration, the decision to migrate is a function of current satisfaction with the profession practised in the home country and that likely to be gained from practising it in that of migration. It seems that the greater the difference in the subjective evaluation of a potential migrant, the higher the likelihood that the intention to migrate will materialize. It should be emphasized that there are various factors underlying this feeling of subjective satisfaction such as the level of remuneration, possibilities of professional development, conditions of work, prestige of the profession, etc. [13]. Each of the abovementioned elements are reflected in the results of work. However, some studies stress that a low salary is the main and strongest factor responsible for professional dissatisfaction among professional nurses. For instance, in the study by Zielinska-Więczkowska [14], the level of remuneration affected professional satisfaction for 98 % of professionally active nurses. The CBOS study [15] also revealed that, in Polish society, professional satisfaction is definitely higher for those with higher incomes (88 %).

According to Leśniowska [16], financial satisfaction is the primary factor responsible for the frequency of decisions made by nurses to seek work abroad. Though decisions to migrate may have a variety of other underlying reasons, comprising the push and pull model [17], it is the difference in socio-economic development, reflected in the stratification of income, that constitutes the main factor underlying migration processes. This view is consistent with the findings herein in which 72.8 % of respondents admitted that they do not find it satisfactory to practise the profession of a nurse in Poland due to their remuneration being too low in comparison with the high level of responsibility and social expectations. The answers given by people declaring the intention to migrate revealed a clear economic character to their lack of satisfaction. This was also evident from the question “*Please, indicate one, most important element which made you consider the country of migration attractive?*” for which the majority of respondents (84/172; 48.8 %) answered “*high earnings*”. Interestingly, the expectations of the majority (48.8 %) of migrating respondents with respect to the remuneration for their work as a nurse abroad seemed to be very realistic [18, 19], as they would be likely to earn an equivalent of PLN 6,500–10,000 ($US 1,722–2,650).

According to Warsaw University researchers studying migration, “… *in the case of migrants with a relatively higher level of education, other rarely analysed factors may be of major importance*” [20]. Indeed, herein, apart from material and living aspects such as high earnings (48.8 %) and better living conditions (22.1 %), the migrating nurses indicated the desire to upgrade their qualifications, i.e. “*better conditions for work and professional development*” (19.2 %) and to gain experience, i.e. “*valuable experience*” (1.7 %), or profession-related prestige, i.e. “*great social respect for the profession of a nurse*” (8.1 %). Yet, non-economic factors play a less significant role as they were indicated by less than one third of the respondents (29.1 %; 50/172).

The relation between the discussed factors observed in our own study should not come as a surprise in the context of Polish nurses, as it is hard to expect them to accept a situation in which their earnings cannot satisfy their basic needs and those of their families. This issue is more evident with data from the Central Statistical Office [21], which indicates that the average gross remuneration among professional nurses and midwives in Poland remains below the average gross salary in the economy as a whole. In the context of push factors, it is worthwhile to draw attention to another, equally important factor affecting professional satisfaction for nurses, which is, namely, inadequate working conditions [22]. Employees tend to perceive that they have an excess workload, which affects the quality and safety of patient care. Understaffing, poor premises, and a lack of basic equipment are some of the problems encountered [23]. Another crucial element is the fact that therapeutic procedures conducted in conditions of permanent underinvestment are encumbered with a higher risk of the occurrence of an adverse event [24].

Regarding barriers to migration, respondents pointed mainly to issues connected with their families (37.6 %); it can be presumed that family issues concern taking care of a sick, disabled, or elderly person. The obvious drawback of the wording of this question was that it was given in a closed instead of an open-ended form. It is consequently impossible to conduct a deep analysis of the reasons for not migrating from the country, which may be only subject to hypothetical considerations in this study. This gap in the present study can be a subject of interest and a point for further research. Other barriers that made respondents prefer to stay in the country, and do not lack clarity, include satisfaction with their own economic situation in Poland (16.8 %) and a lack of knowledge of foreign languages (16.6 %). The latter is most likely to lose significance with future generations of young nurses due to the ever-growing level of education in Polish society [25]. Apart from the level of education, there are also other features favouring mobility that have been indicated by numerous researchers, such as age or willingness to take a risk [20, 26].

Destinations of migration (mainly Germany and England) indicated by the respondents come as no surprise with the available Central Statistical Office data concerning nationwide migration trends registered in Poland [27, 28]. It can be supposed that the countries referred to were indicated by the respondents due to the reasons of migration specified in an earlier question – that is, mainly economic considerations. This is consistent with the fact that these countries are known to be relatively rich countries [29]. Caution, however, should be taken in making such generalizations. As it has already been mentioned above, the decision to migrate is a product of a number of factors. Though economic factors play a crucial role in a decision to migrate, the migration theory of Lee [12] indicates that personal factors can underpin the decision to choose a particular country. A similar position can be found in the theory of migration networks, which similarly notes that the choice of a particular country can be affected by existing social links as these can lower migration (including psychological ones) and accommodation-related costs, making the country a more attractive destination [12].

Long-term migration (over a year, according to the International Organization for Migration) [30] seemed to be a prevailing desire among the respondents. Over one third of respondents intended to leave the country for a period ranging from 2–5 years. More worryingly, a similar percentage of respondents intended to leave Poland permanently. This is a particularly painful loss, not only for the local community where a given nurse worked, but also for the society as a whole when considered in terms of the cost of educating a given person, in the case of a public financing system.

Respondents (106/581) who declared migration for a specified period of time indicated, most frequently, that they intend to re-migrate to the same country as before. This is consistent with respondents’ answers to the question concerning destinations of migration, as they chose countries close to Poland (e.g. Germany, England, or Norway), the choice being perhaps dictated by the desire to save time in the case of frequent travels between countries.

The most frequent place of work once outside Poland indicated by the largest number (50.0 %) of respondents were hospitals. It should, however, be kept in mind that in the near future, the greatest number of jobs will be in healthcare centres and facilities providing nursing and therapeutic care catering for the needs of the elderly, as driven by the growing elderly population and the change in the structure of diseases (co-existence of chronic age-related diseases and sensory organ dysfunction) [31, 32]. An increased consumption of health, nursing, and care services will follow [33]. Consequently, the healthcare system will be oriented towards patients who live longer but have limited or no independence and thus require long-term care [34]. Long-term care is a strategic area for the healthcare market in the EU as it is likely to generate an increased demand for medical personnel [35]. For instance, in Poland, this would be because of the decreased care potential of the family (low birth rate, one-person households, family migrations, weakening of family ties), among other factors. The number of non-medical caretakers is likely to decrease and care of the elderly will be taken over by institutions [10].

Nursing homes and other institutions taking care of the elderly were the second most common setting to be indicated by the respondents (27.3 %) as their first place of work. In addition, among respondents who intend to migrate, the majority (89.5 %) planned to migrate and work in accordance with their qualifications as a nurse, while almost one in ten would take up work abroad below their qualifications, primarily as a caretaker of an elderly person. The question did not specify why, but it can be assumed to be due to a lack of sufficient command of the local language.

What should be said and remembered about the results of the present study is the fact that they refer only to the studied group of nurses. An attempt to generalize the presented findings and extend them to a broader population of nurses does not seem justified, despite the large size of the sample and the fact that the destinations for migration of the nurses studied corresponded with those from Central Statistical Office data concerning the migration of Polish citizens. Given the way the sample was collected and the use of the Internet as a means to reach the respondents, it is impossible to estimate the correctness and applicability of the selected data for the overall population of Polish nurses. The obvious merit of the study is no doubt its pioneering character in the area of Polish nurse migration, and consequently the study constitutes a valuable starting point for further research in the area from a methodological point of view.

Efficient migration management in healthcare seems to be a strategic factor that can contribute to the effective functioning of the healthcare system as a whole in light of the low level of human resources in the Polish nursing sector and the demographic changes taking place [36].

It is worth mentioning that the results of the study are consistent with the recommendations of the World Health Organization contained in its Global Code of Practice on the International Recruitment of Health Personnel in “*Article 6 – Data gathering and research*”. It was written that Member States should be committed to the following principles: “(6.1) *effective policies and plans on the health workforce requires a sound evidence base*, (6.2*) establish or strengthen and maintain, as appropriate, health personnel information systems, including health personnel migration, and its impact on the health system*, (6.3) *establish or strengthen research programmes in the field of health personnel migration and coordinate such research programmes through partnerships at the national, subnational, regional and international levels*, (6.4) *in collaboration with WHO, relevant international organizations and Member States, ensure, as much as possible, that comparable and reliable data are generated and collected pursuant to paragraphs 6.2 and 6.3 for ongoing monitoring, analysis and policy formulation*” [37].

## Conclusions

Analysis of intentions to migrate abroad in the studied group of Polish nurses shows that there is great interest in seeking employment abroad consistent with their qualifications, that is, as a hospital nurse or in institutionalized nursing care units for the elderly. The nurses indicated an intention to migrate mostly for repeated long-term stays or a permanent stay to Germany, a country of migration that is already a long tradition among Poles, or to England or Norway. The level of earnings expected by the nurses is in compliance with the actual level of earnings in the countries in question, which seems to be evidence of their serious interest in migrating. Given the methodology applied, study findings refer solely to the study group.

There is an undeniable need for long-term actions to improve the working conditions and remuneration of Polish nurses. If this does not occur, the human resource crisis in the Polish nursing sector will deepen. Referring to the official estimation of the Supreme Chamber of Nurses and Midwives, If 60,000–80,000 nurses in the Polish system were to retire in the near future, Poland will have serious problems in coping with a nursing shortage. In view of the low level of human resources in the Polish nursing sector, the migration of Polish nurses will probably have crucial implications for maintaining the standard of healthcare in Poland in the coming years.
